# Positive experiences of volunteers working in deployable laboratories in West Africa during the Ebola outbreak

**DOI:** 10.1371/journal.pone.0196320

**Published:** 2018-04-26

**Authors:** Evelien Belfroid, Madelief Mollers, Pieter W. Smit, Marlies Hulscher, Marion Koopmans, Chantal Reusken, Aura Timen

**Affiliations:** 1 National Institute for Public Health and the Environment (RIVM), Centre for Infectious Disease Control, Preparedness and Response Unit, Antonie van Leeuwenhoeklaan 9, Bilthoven, The Netherlands; 2 Radboud University Medical Center, Radboud Institute for Health Sciences, IQ healthcare, Nijmegen, The Netherlands; 3 Erasmus MC, Department of Virology, WHO Collaborating Centre for Arbovirus and Hemorrhagic Fever Reference and Research, Rotterdam, The Netherlands; Instituut voor Tropische Geneeskunde, BELGIUM

## Abstract

The largest outbreak of Ebola virus disease ever started in West Africa in December 2013; it created a pressing need to expand the workforce dealing with it. The aim of this study was to gain insight into the experiences of volunteers from the European Union who worked in deployable laboratories in West Africa during the outbreak. This study is part of the EMERGE project. We assessed the experiences of 251 volunteers with a 19-item online questionnaire. The questions asked about positive aspects of volunteering such as learning new skills, establishing a new path in life, and changing life values. Other questionnaire subjects were the compliance to follow-up measures, the extent to which volunteers felt these measures restricted their daily activities, the fear of stigmatization, and worries about becoming infected or infecting their families. The volunteers reported positive effects that reached far beyond their daily work, such as changes in life priorities and a greater appreciation of the value of their own lives. Although the volunteers did not feel that temperature monitoring restricted their daily activities, full compliance to temperature monitoring and reporting it to the authorities was low. The volunteers did not fear Ebola infection for themselves or their families and were not afraid of stigmatization. With respect to the burden on the families, 50% reported that their family members were worried that the volunteer would be infected with Ebola virus. Altogether, the positive experiences of the volunteers in this study far outweigh the negative implications and constitute an important argument for inspiring people who intend to join such missions and for motivating the hesitant ones.

## Introduction

The largest outbreak of Ebola virus disease to date originated in West Africa in December 2013. It caused 28,616 Ebola cases in Guinea, Liberia, and Sierra Leone, which resulted in 11,310 deaths [1]. It takes more than 25 healthcare workers to care for 10 Ebola patients, meaning that thousands of healthcare workers and volunteers were needed to contain the outbreak [2]. As the Ebola outbreak unfolded to an unprecedented magnitude, a large demand on human resources that included physicians, nurses, and laboratory staff emerged [2].

The severity of the disease, the lack of curative treatment, and the high risk of contamination were major challenges. Healthcare workers from western countries were hesitant to volunteer or did not find suitable channels through which to do so [3, 4]. Of all the local healthcare workers in West Africa, 1.4–8.7% died of Ebola, compared to 0.02–0.11% of the general population [5]. Due to the uncontrolled spread of the disease locally and the media’s alarming reports about cases imported into Europe and the USA, many governmental and non-governmental organizations worldwide, along with military personnel, finally provided support to the most affected countries. Trained staff, equipment, and facilities for treatment and testing were deployed or set up in West Africa. At present, there is still very little insight into the experiences of the volunteers who worked in this high-risk setting. The few studies on healthcare workers volunteering during the Ebola outbreak describe the barriers and facilitators that healthcare workers encountered when they consider to volunteer [3, 4].

Although diagnostic laboratory work can be considered less dangerous than patient care because there is no direct patient contact, anxiety still caused difficulty in the enrolment of personnel. This is likely due to the fact that laboratory personnel processed patients’ specimens that were positive for Ebola virus and were aware that any breach in observing strict laboratory precautions might lead to exposure and infection. Insight into the experiences of volunteers who actually did go to West Africa is needed to learn the lessons that will prepare volunteers more adequately to participate in future emergency workforce teams.

This study reports on the personal experiences of European Union (EU) volunteers deployed in laboratories in West Africa during the Ebola outbreak that began in 2013. Mobile laboratories were often located at Ebola treatment units; laboratory staff had close contacts with the clinical staff.

## Methods

This study is part of the EMERGE Joint Action project. The general objective of EMERGE Joint Action is to enable an efficient response to serious emergent and re-emergent cross-border events by reinforcing the existing EU network of Biosafety level 3 and Biosafety level 4 laboratories that are already actively identifying dangerous human pathogens of the bacterial and viral types. An online questionnaire was sent to the laboratory personnel who were voluntarily deployed by European organizations to work in West Africa during the Ebola outbreak. This questionnaire evaluates technical aspects of laboratory preparedness and deployment. We augmented it with a subset of questions that ask about well-being and the personal experiences of volunteers during and after the mission. We report on the results of the subset questions and highlight the implications for future missions.

### Study population

Eight organizations, which provided 17 associated mobile laboratories, were involved in sending out volunteers. We emailed the questionnaire to 646 volunteers who were deployed by six of the eight organizations (the six had 14 of the 17 European mobile laboratories). After emailing them in January 2016, we repeated our message with reminders for those who had not responded. The online questionnaire was opened by 302 recipients and was completed by 251 respondents (response rate: 38.9%, completion rate: 83%). The median time between deployment (the first day of the initial month of deployment) and the time of completing the questionnaire was 373 days, range 82–772). Any volunteer who had been on multiple missions was asked to report on only one mission. The last questionnaire was completed by July 1, 2016. The Clinical Expertise Centre of the National Institute for Public Health and the Environment reviewed the study protocol (LCI-350). On the basis of this review, they determined that the research plan did not fall under the Dutch law on medical research involving humans (WMO). All the respondents consented to participate in the study and were aware that their responses would be used for research purposes. Data were collected anonymously.

### Questionnaire design

There were 19 statements (Table 1) about aspects of volunteering such as learning new skills, establishing a new path in life, changing life values, compliance with follow-up measures, the extent to which volunteers felt these measures restricted their daily activities, and worries about being infected or infecting their families. We asked the respondents to assess their fear of stigmatization after they returned from the mission.

**Table 1:** Volunteer experiences.

Domain	Statement	0 Strongly disagree (%)	1 (%)	2 (%)	3 Neutral (%)	4 (%)	5 (%)	6 Strongly agree (%)
**Experience**	This experience provided me with a sense of meaning and purpose	0	0.8	0.4	4.8	12.4	24.1	57.4
	I have learned new skills that I can use in my current and/or future job	0.4	0.4	2.4	9.2	21.3	22.1	44.2
	I would go on a mission again	0	0	0.8	2.4	8.8	20.1	67.9
	I have changed my priorities about what is important in life	2.4	2.4	2.8	24.5	28.1	18.1	21.7
	I have a greater appreciation of the value of my own life	2.0	2.8	2.4	28.5	21.3	18.9	24.1
	I have a greater sense of closeness with others	4.8	3.2	4.4	36.1	26.9	13.7	10.8
	I can do better things with my life	3.6	4.0	2.8	38.0	23.6	15.6	12.4
	I have established a new path for my life	9.2	7.2	6.0	39.4	19.7	8.8	9.6
	I know better that I can handle difficulties better than before this deployment	3.2	2.4	4.0	22.4	20.4	25.6	22
	I have discovered that I am stronger than I thought I was	4.0	4.4	3.2	30.4	20	17.6	20.4
**Follow-up measures**	I fully complied with temperature monitoring for 21 days after my return	4.0	3.2	5.6	24.5	7.2	11.2	44.2
	Monitoring my temperature made me feel restricted in my daily activities	48.8	17.7	8.9	21.8	0.8	0.8	1.2
	I reported my temperature to the authorities concerned	38.1	4.0	2.0	29.6	4.9	2.4	19.0
**Worries**	I was worried about being infected with the Ebola virus	37.3	25.3	10.8	16.5	6.4	2.4	1.2
	I was worried about infecting my family or others with the Ebola virus	46.8	20.4	8.0	14	8.0	1.2	1.6
	My family were worried about my being infected with the Ebola virus	11.7	8.1	9.3	20.6	29.6	9.7	10.9
	My family were worried about being infected with the Ebola virus themselves	24.6	18.1	12.5	24.6	12.9	3.6	3.6
**Stigma**	I was afraid of being stigmatized after my return	43.8	14.5	8.8	18.1	10.4	1.6	2.8
	My family were afraid of my being stigmatized after my return	55.4	16.9	6.4	14.9	3.2	1.6	1.6

One to four items of data were missing from each statement in this table

The questionnaire was based on questionnaires that had been used previously to collect the experiences of healthcare workers caring for two patients with Middle East respiratory syndrome and a case of Marburg hemorrhagic fever in the Netherlands [6, 7]. Items from the Post-traumatic Growth Inventory were used as well [8]. We asked the respondents to what extent they agreed with the statements included in the questionnaires on a 7-point Likert scale (0 thru 6; 0 = strongly disagree, 3 = neutral, 6 = strongly agree).

### Analysis

We used our descriptive analysis of the data and a linear regression to study the relation between the sum score of each domain versus the age group and country where the volunteer worked. We calculated the sum scores by adding up the scores within each domain.

## Results

### Study population

Of the 251 volunteers, 162 (65%) volunteered to go to Sierra Leone, 76 (30%) went to Guinea, and 13 (5%) went to Liberia. The first 70 participants (28%) volunteered in 2014, and the remaining 180 (72%) volunteered in 2015. Most volunteers (45%) were 26 to 35 years old and 27% were 36 to 45 years old.

### Questionnaire: Volunteers’ experiences

**Fig 1:**
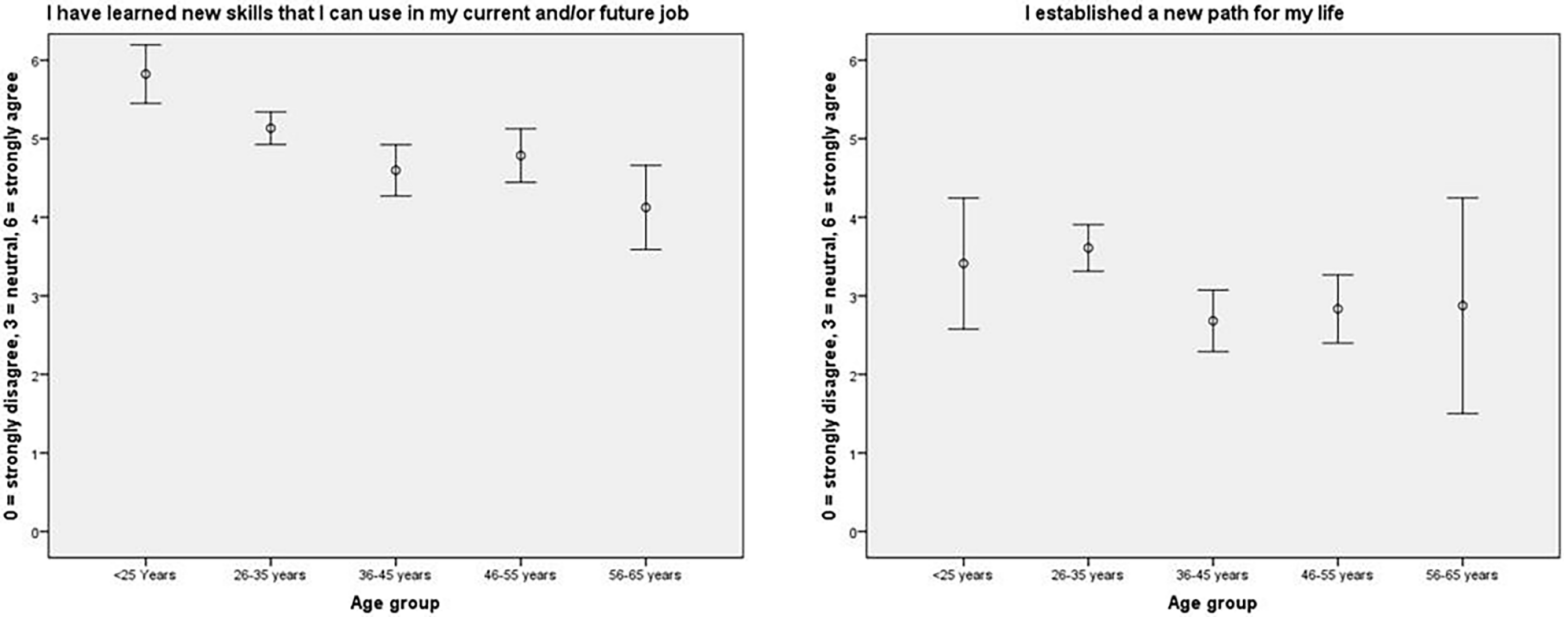
Mean and 95% confidence interval per age group.

The results in this paragraph are the perceived results of the volunteers’ experiences in the Ebola virus disease outbreak. Overall, the respondents were very positive about volunteering in a high-risk setting. Most volunteers (234 of 249, 94%) stated that the experience provided them with a sense of meaning and purpose, and almost 97% stated that they would go on a mission again. The volunteers felt a greater sense of closeness with others (51%), knew that they could handle difficulties better (68%), and discovered they were stronger than they thought they were (58%). In addition, the volunteers thought that they had a greater appreciation of the value of their own lives (64%) and that they had changed their priorities about what is important in life (67.9%). There were differences among the age groups (<25 years, 26–35 years, 36–45 years, 46–55 years, and 56–65 years) for the items “I have learned new skills that I can use in my current and/or future job” and “I established a new path for my life” (Fig 1). The two youngest age groups were better at learning new skills than the oldest age group. Volunteers aged 26–35 years were better at establishing a new path for their lives than volunteers aged 36–55 years. We found no differences between the age groups for the other items in this domain.

### Relation between the domains and the age groups

Because only 13 volunteers went to Liberia, we had to exclude this group from our analysis. We aggregated the age groups into three groups (≤35 years, 36–45 years, and 46–65 years) to get closer to equal numbers. We calculated the sum scores of each domain by adding up the scores of the variables corresponding to each domain. For the domain “follow-up measures”, we recoded the variable “monitoring my temperature made me feel restricted in my daily activities” so that all variables in the domain represent a positive trend. We found no significant results by linear regression in the relation between the sum scores of the domains versus age and country except for the domain “experience”. We found a difference of -3.89 between the youngest and oldest age groups (*p* = 0.044) and a difference of -6.40 between the youngest group and the middle-aged group (*p* = 0.001). This means that younger volunteers were the most positive about the experience and volunteers aged 36–45 years were the least positive. We found no significant difference for the country the volunteer was sent to.

### Follow-up measures

All volunteers had to monitor and report their temperature for the 21 days following the last exposure, regardless of whether they had a fever. Forty-four percent (score = 6) of the respondents said that they had fully complied with temperature monitoring, and 75% of the respondents did not feel that monitoring their temperature restricted their daily activities. Almost 13% did not fully comply with temperature monitoring for the 21 days and 3% of the respondents felt that monitoring their temperature restricted their daily activities. Over 26% of the respondents reported their temperature to the authorities in charge. We found no differences between the five age groups.

### Worries and stigma

Ten percent of the respondents said they were worried about being infected with the Ebola virus, and 11% were worried about infecting their family members with the Ebola virus. Half of the respondents (50%) agreed that “My family members were worried that I would be infected with the Ebola virus”, and 20.1% agreed that “My family members were worried about being infected with the Ebola virus.” Almost 15% were afraid of being stigmatized after their return, and 6.4% of the respondents’ families were afraid they would be stigmatized. We found no differences between the five age groups.

## Discussion

This study captures the experiences of volunteers who worked in a high-risk setting during the Ebola virus disease outbreak. Volunteering for such a mission can have major implications for the volunteers themselves and for their families [9–11]. The volunteers report positive experiences that reached far beyond their daily work, such as a change in their priorities in life and a greater appreciation of the value of their own lives. Although the volunteers reported that temperature monitoring did not make them feel that their daily activities were restricted, fully complying with temperature monitoring and reporting it to the authorities was low. The volunteers did not fear infection with Ebola or infecting their families, and they were not afraid of being stigmatized. With respect to the burden on family members, 50% reported that their family members were worried that the volunteer would be infected with Ebola virus.

Our paper shows that those who did volunteer derived a lot of satisfaction from their experiences. Most of our respondents were not worried about being infected with Ebola or infecting family members. Nevertheless, their families were concerned. This effect has also found in other studies, such as the one reporting on a patient with Marburg hemorrhagic fever. In that study, 40% of the 130 contacts reported that their family members expressed anxiety about becoming infected, and over 30% reported that their partners were disturbed by the restrictive control measures [7]. In another study of the experiences of Dutch healthcare workers caring for a suspected Ebola patient, we found that family members of healthcare workers were afraid and anxious [12]. One explanation may be that healthcare workers are better informed about the risks and protective measures than their family members are, which reduces worry and anxiety. However, family members’ fears and anxieties can influence the volunteer’s decision to participate in such a mission. For those volunteering in the event of future outbreaks, it is important that the families’ worries are addressed at an early stage of the recruitment.

In our study, almost half of the respondents complied with daily measuring, but we notice a low reported compliance with daily reporting of temperature to the authorities. Over 26% of the volunteers reported their temperature to the authorities in charge. Compared to other studies (adherence rates ranging from 80 percent in the first weeks after exposure to 66% after 6 weeks of exposure) this is low [7]. The reasons for that have not been asked, but there might have been difference with respect to how authorities required reporting between the countries sending volunteers. This is an aspect that deserves attention when developing instruction for volunteers in future missions.

The reported perceptions of risk in this study are low. However, the actual risks are also likely to be low for a well-trained and experienced volunteer. Thus, we believe that the perceived risks represent the actual risks.

Almost half of our respondents were between 26 and 35 years old. This is similar to the findings in Turtle’s study in which 43% of those considering volunteering were in the 26–35 year age group [4]. In recruitments for future outbreaks, most of the volunteers will be less than 35 years old. Volunteers of this age reported the most positive experiences in our study. This might be explained by the assumption that younger people are more adventurous or do not have children and are therefore more inclined to work in a high-risk environment. A previous study showed that potential volunteers without children were less afraid of contracting Ebola virus disease [4]. This age group might be considered a primary target for volunteer missions, while it must be taken into account that senior experts are also needed because they have the necessary hindsight and expertise; they provide guidance and support to younger volunteers and help constitute a well-balanced group of volunteers.

This study has some limitations. First, there may be recall bias, given the retrospective nature of the study. Second, only a minority of all the volunteers who were contacted completed the questionnaire. Volunteers who experienced the mission as positive might have been more prone to complete the questionnaire, causing selection bias. However, in our previous study in which we interviewed healthcare workers who cared for a patient suspected of Ebola virus disease healthcare workers also reported positive experiences [12]. Third, the volunteers reported their family members’ experiences. Firsthand information from relatives and family members themselves would help us to gain more insight into their actual needs. The questionnaire did not provide data from people who wanted to volunteer but did not because their families did not want them to. Bearing in mind the expected need for volunteers for future outbreaks, their insights and needs would also be very valuable. Fourth, respondents could decide for themselves which mission they wanted to report. Respondents who went on multiple missions may have reported more positive experiences than volunteers who went on one mission only because they were more experienced and accustomed to the situation. However, the setting was identical.

The response rate for this study was 38%. Several factors may explain our response rate. First, the time span between the actual mission and receiving the questionnaire might have had a negative impact on the response rate. Second, volunteers might have already talked through their experiences with their organization or their family and friends and did not feel the need talk about their experiences again. Given the knowledge gap in the literature about the experiences of volunteers working in high-risk environments, we believe that our study is very valuable.

Our study reports on personal experiences systematically collected from 251 volunteers who engaged in circumstances that were distinct from their daily routines. They were required to carry out new tasks in an unusual situation associated with significant risks, and they had to work in a new team. The Ebola crisis made it clear that there was a need for a European Medical Corps and a global health emergency workforce for strengthening the emergency capacity and the international capability to respond to crises. Both of these are now installed [13–15]. The results of our study could be used as a starting point to aid the workforce in preparing healthcare workers for their tasks. It must be kept in mind that healthcare workers, while working in potentially even more dangerous environments than laboratory workers, might have experiences similar or less positive than our volunteers. Special attention should be paid to the fear and anxiety of the healthcare worker’s family, both of which need to be addressed beforehand. Altogether, the positive experiences of the volunteers in this study far outweigh the negative implications. These experiences may constitute an important argument for inspiring people who intend to join such missions and to motivate the hesitant ones. For most respondents, participating in this volunteer mission resulted in an immaterial long-term yield of new experiences and a purposeful meaning of life.
